# Fibrin γ/γ' influences the secretion of fibrinolytic components and clot structure

**DOI:** 10.1186/s12860-019-0233-0

**Published:** 2019-11-01

**Authors:** Miriam Cantero, Héctor Rojas, Eduardo Anglés-Cano, Rita Marchi

**Affiliations:** 1 Departamento de Bioquímica, Universidad de Córdoba, Córdoba, Colombia; 2 Centro de Medicina Experimental, Laboratorio Biología del Desarrollo de la Hemostasia, Instituto Venezolano de Investigaciones Científicas, Caracas, Venezuela; 3 Instituto de Inmunología, Universidad Central de Venezuela, Caracas, Venezuela; 4 Université de Paris, Innovative Therapies in Haemostasis, INSERM, F-75006 Paris, France

**Keywords:** Fibrinogen gamma prime (γ’), Clot structure, Urokinase plasminogen activator (uPA), Plasminogen activator inhibitor type 1 (PAI 1), Human dermal microvascular endothelial cells (HMEC-1)

## Abstract

**Background:**

In healthy subjects fibrinogen γ/γ‘ circulates at 8–15% of the total plasma fibrinogen concentration. Elevated levels of this variant have been associated with arterial thrombosis, and its diminution with venous thrombosis. The aims of the present work were to analyze the structure of the fibrin network formed on the top of human dermal microvascular endothelial cells (HMEC-1) at different fibrinogen γ/γ‘ concentrations, as well as its influence on the secretion of fibrinolytic components.

The kinetics of fibrin polymerization on top of HMEC-1 cells with 3, 10, and 30% fibrinogen γ/γ‘ was followed at 350 nm. The secretion of urokinase-type plasminogen activator (uPA) and plasminogen activator inhibitor type 1 (PAI 1) by HMEC-1 were measured in the supernatant and cell lysates, after incubation with 1 nM thrombin, fibrin with 3, and 30% fibrinogen γ/γ‘, using commercial kits. The influence of fibrinogen γ/γ‘ on fibrin structure on the surface of the HMEC-1 was followed with laser scanning confocal microscopy (LSCM).

**Results:**

The kinetics of fibrin formation on HMEC-1 with 3 and 10% fibrinogen γ/γ‘ were similar. However, with 30% fibrinogen γ/γ‘ both the slope and final turbity were approximately 50% less. The LSCM images showed the dramatic effects of increasing fibrinogen γ/γ‘ from 3 to 30%. The uPA and PAI 1 concentrations in culture supernatants HMEC-1 cells treated with thrombin or 30% γ/γ‘ fibrin were two-fold increased as compared to basal culture supernatants and 3% γ/γ‘ fibrin-treated HMEC-1. In all stimulatory conditions the intracellular concentration of uPA was higher than in supernatants. In contrast, the intracellular PAI 1 concentration was decreased as compared to that measured in the supernatant, including the basal condition.

**Conclusion:**

A concentration of 30% fibrin γ/γ‘ alter drastically fibrin structure on the cell surface and affects the secretion of uPA and PAI 1 through its capacity to bind thrombin.

## Background

Fibrinogen is a soluble plasma protein that polymerizes spontaneously upon cleavage of fibrinopeptides A and B by thrombin. The protein is made up of six polypeptide chains Aα, Bβ and γ, arranged as a dimer. Fibrinogen comprises a heterogeneous population of molecules. The γ chain has two isoforms, the most abundant form, denoted as γ or γA, represents approximately 80–85% of the total fibrinogen and is homodimeric [1, 2], and the minor isoform denoted as γ’ [1] arises from alternative splicing in intron 9, resulting in the loss of exon 10 and the retention of part of intron 9 in the mRNA [3]. This mRNA translate a new extended γ carboxy-terminal of 20 amino acids VRPEHPAETEYDSLYPEDDL (γ’408–427) not present in the most abundant isoform (γ 408–411; AGDV) [4]. The γ’ chain is present in fibrinogen mostly as a heterodimer (γ/γ’) and less than 0.5% as homodimer (γ’/γ’) [1, 5]. The γ’ chain introduces negative charges to fibrinogen due to the abundance of acidic amino acids and the sulphation of tyrosine residues, with pronounced effects on fibrin (ogen) functions and structure. Fibrinogen participates in primary hemostasis by bridging platelets. The carboxy-terminal of γ chain accomplishes this task through the binding of the AGDV sequence (408–411) to the α_IIb_β_3_ (GpIIb-IIIa) platelet receptor [6]. Because the AGDV stretch is replaced by 20 news amino acids in γ’, this chain does not binds to platelet, and thrombin-induced platelet aggregation is reduced [7].

Fibrinogen γ’ binds thrombin and FXIII [8]. The binding of thrombin to γ’ reduce its availability in solution [9], and protect its activity from inhibition by antithrombin and heparin [10]. The effects of the binding of γ’ chain to the FXIII B subunit are controversial [11]. Moaddel et al. found that FXIII binds to fibrinogen γ/γ’ with higher affinity compared to fibrinogen γ/γ, and that fibrinogen γ/γ’ had a greater effect on the enhanced activation kinetics of FXIII [12]. However, Gersh and Lord did not find differences [13]. Additionally, Moaddel et al. reported that fibrinogen γ/γ’ cross-linked faster and to a greater extent [12]. However, Siebenlist et al. found that fibrinogen γ/γ’ and plasma FXIII complex down regulates the activity of plasma FXIII crosslinking [14].

Moreover, fibrinogen γ’ has an anticoagulant effect by diminishing FVIII and FV activation, and increases plasma activated protein C sensitivity [15–17].

The clot architecture is influenced by fibrinogen and thrombin concentrations, ionic strength and pH [18] as well as binding and crosslinking of different plasma proteins [19]. Several groups have shown that clots formed with fibrinogen γ’ have thinner fibers and small pores [20, 21], decreased stiffness [22], and are more resistant to lysis [23]. Considering the structural and functional modifications introduced by this minor γ chain isoform, it is not surprising that fibrinogen γ’ has emerged as a risk factor for cardiovascular disease [24]. The reports of biochemical changes induced in cultured endothelial cells by fibrin [25, 26], led us to hypothesize that fibrinogen γ’ chain may influence fibrin polymerization and endothelial cell secretion of fibrinolytic components. The aim of the present work was therefore to determine whether different proportions of γ’ chain influences fibrin polymerization on model endothelial cells (the human microvascular endothelial cell line, HMEC-1) that might affect the secretion of the plasminogen activator synthesized by HMEC-1 (uPA) and its inhibitor PAI 1.

## Results

### Fibrin formation on HMEC-1

**Fig. 1:**
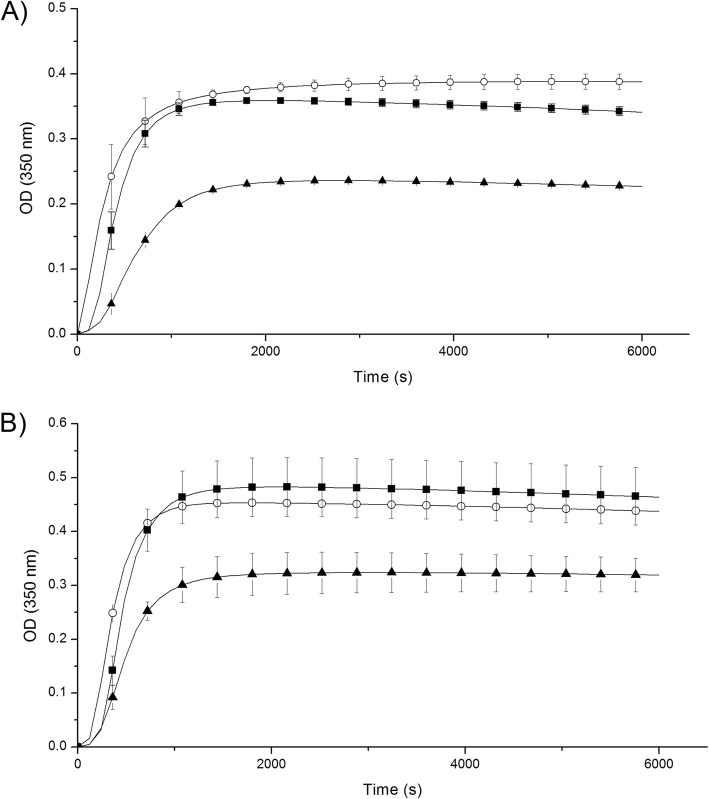
Kinetics of fibrin formation at different fibrinogen γ/γ’ concentrations. Clots were formed by mixing purified or commercial fibrinogen γ/γ and γ/γ’ with 1 nM thrombin and 2 mM CaCl_2_, final concentrations. The OD changes were recorded at 350 nm each 2 min, during 1 h 40 min. **A** Clot formed on the top HMEC-1. **B** Clot formed without HMEC-1. Fibrinogen γ/γ’ 3% (◯); fibrinogen γ/γ’ 10% (■); fibrinogen γ/γ’ 30% (▲)

The kinetics of fibrin formation at 3 and 10% fibrinogen γ/γ’ in the presence of HMEC-1 cells were similar to those without cells (Fig. 1; Table 1). However, the lag time was lengthened in the presence of fibrinogen γ/γ’ higher than 3%, regardless of the presence of cells. Clots formed with 30% of fibrinogen γ/γ’ were less turbid, and the rate of fibrin formation slower as compared to clots formed with 3 and 10% fibrinogen γ/γ’ (*p* < 0.05), with or without cells (Fig. 1). In the presence of cells, the fibrin formed with 30% fibrinogen γ/γ’ increases 2-times the MaxAbs and 1.4-times the slope compared to the fibrin formed with 30% fibrinogen γ/γ’ without cells.

**Table 1:** Summary of fibrin polymerization kinetics at different fibrinogen γ/γ’ concentrations on HMEC-1. Results are expressed as mean ± standard error (SE). Experiments were performed each time in triplicate in three independent experiments

γ/γ’ (%)	MaxAbs (mOD)		Slope (mOD/min)		LT (min)	
	+Cell	-Cell	+Cell	-Cell	+Cell	-Cell
3	440 ± 20	390 ± 10	60 ± 2	45 ± 10	0	0
10	460 ± 50	340 ± 30	60 ± 5	49 ± 6	3	3
30	320 ± 30*	230 ± 30*	36 ± 2*	20 ± 6*	3	3

(+) Cell: in the presence of HMEC-1 cells; (−): in the absence of HMEC-1 cells

*p < 0.05: Fibrin formed in the absence or on HMEC-1 culture at 30% γ/γ’,

compared to that of 3 and 10% γ/γ’

### HMEC-1 uPA and PAI 1 secretion under different stimulation conditions

The amounts of uPA secreted by HMEC-1 in the presence of thrombin or 30% fibrin γ/γ’ were similar, and approximately 2-times greater than that secreted under basal conditions or in the presence of 3% fibrin γ/γ’ (*p* < 0.05) (Table 2). The uPA concentration inside the cells was 4, 6 and 8-times higher in the presence of 3% fibrin γ/γ’, thrombin and 30% fibrin γ/γ’, respectively, compared to basal condition (Table 2).

**Table 2:** Effect of fibrin γ/γ’ on secretion of uPA and PAI 1 by HMEC-1. Results are expressed as mean ± standard error (SE). Experiments were performed each time in triplicate in three independent experiments

	uPA		PAI 1	
	SN	CL	SN	CL
Basal	0.56 ± 0.09	0.40 ± 0.01	2569 ± 344	1055 ± 85
3% γ/γ’	0.39 ± 0.13	1.73 ± 0.11**^ξ^	3398 ± 436	770 ± 74
30% γ/γ’	1.06 ± 0.04*	3.21 ± 0.03**	5976 ± 918*	1845 ± 201*
Thrombin	1.04 ± 0.09*	2.42 ± 0.48**	6464 ± 455*	1422 ± 229*

SN: supernatant; CL: cell lysate (1000 cells/μL). uPA and PAI 1 concentrations are in ng/mL

*p < 0.05 when compared to the basal condition and 3% fibrin γ/γ’

**p < 0.05 when compared to the basal condition

^*ξ*^*p < 0.05* when compared to 30% fibrin γ/γ’

The PAI 1 secreted to the medium was 1.3, 2.3 and 2.5 times higher in 3% γ/γ’, 30% γ/γ’ and thrombin, respectively, compared to the basal condition (Table 2). The PAI 1 stored inside the cell increased significantly in the presence of thrombin alone or 30% γ/γ’ (1.4-times and 1.7-times, respectively; Table 2).

**Fig. 2:**
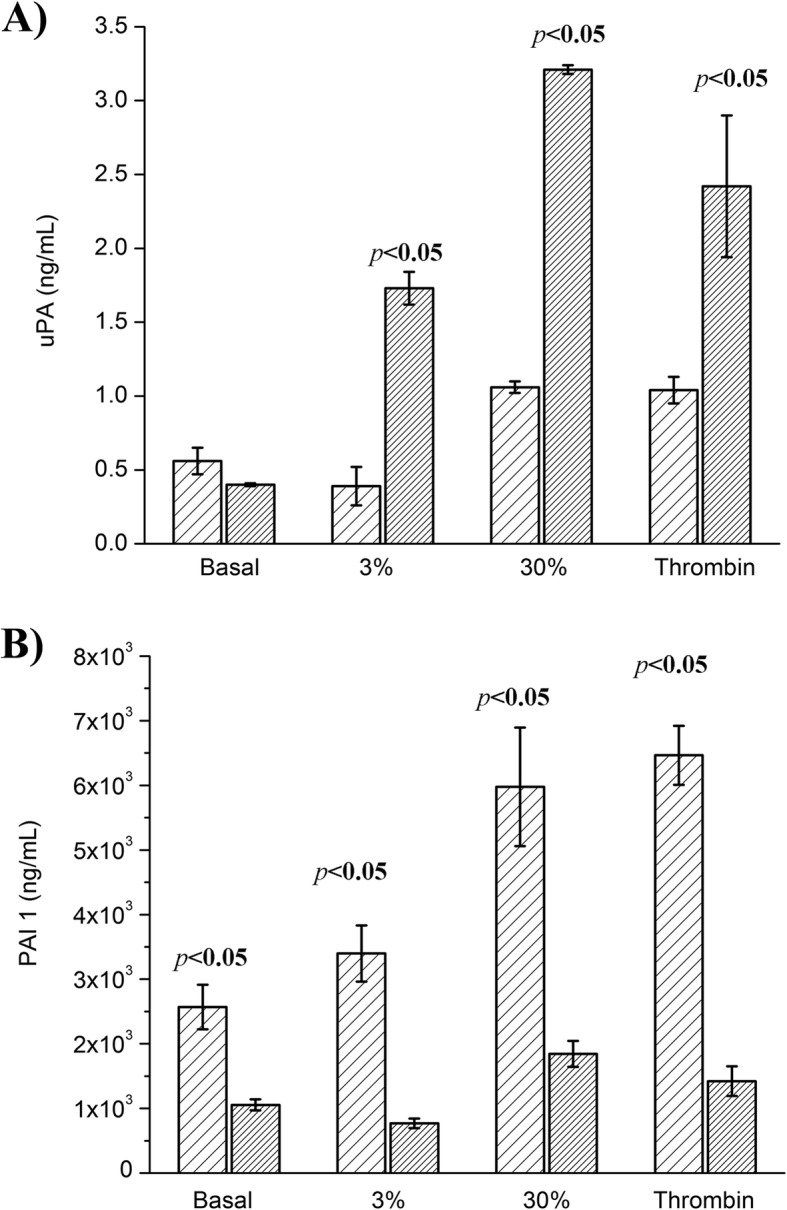
uPA and PAI 1 concentration under different stimulation conditions. The basal and thrombin condition consisted of HMEC-1 culture incubated with medium + 10% FBS and 1 nM thrombin and 2 mM CaCl_2_, respectively. The 3 and 30% fibrin γ/γ’ conditions consisted of HMEC-1 culture incubated with clots formed with purified fibrinogen (3% γ/γ’) and 30% fibrinogen γ/γ’ (by mixing commercial fibrinogen γ/γ and γ/γ’). A) uPA (ng/mL) and B) PAI (ng/mL) quantified by ELISA in the supernatant () and cell lysate (). The uPA concentration in the supernatant of basal condition was similar to that of cell lysate, while those of thrombin, 3 and 30% fibrin γ/γ’ were significantly higher in the cell lysate (*p* < 0.05). Instead, the PAI concentration was higher in the supernatant in all stimulatory and basal conditions (*p* < 0.05)

The comparison between the extra- and intracellular uPA and PAI 1 concentrations are shown in Fig. 2. The uPA secreted to the medium was lesser compared to that present inside the cells for 3% γ/γ’, 30% γ/γ’, and thrombin (*p* < 0.05) (Fig. 2a). In contrast, the PAI 1 concentration was higher in the supernatant compared to that inside the cells in all the conditions used (p < 0.05), (Fig. 2b).

### Expression of uPA receptor by HMEC-1

**Fig. 3:**
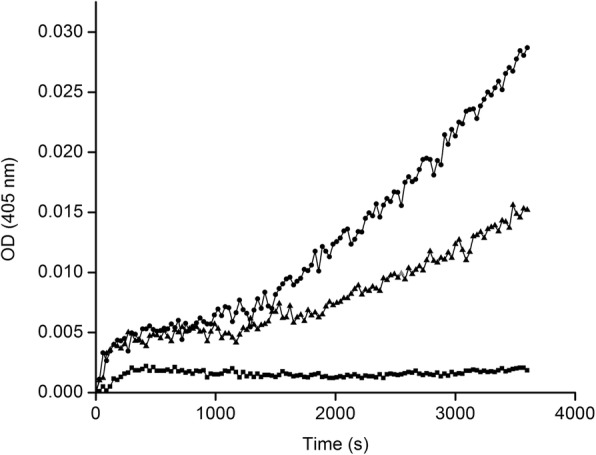
Presence of uPAR on HMEC-1 cells. HMEC-1 cells were incubated with uPA, then with plasminogen. The plasmin activity was revealed with chromogenic substrate S-2251. (■) Without uPA; (▲) 10 nM uPA; and (●) 20 nM uPA

The strategy used to measure the presence of the receptor was its capacity to bind uPA. Receptor-bound uPA was then detected by its ability to convert plasminogen into plasmin, which was detected with the chromogenic substrate S-2251. A representative experiment is shown in Fig. 3, at 10 and 20 nM uPA, demonstrating the presence of uPA receptors at the plasma membrane of HMEC-1 cells.

### Fibrin interaction with HMEC-1

**Fig. 4:**
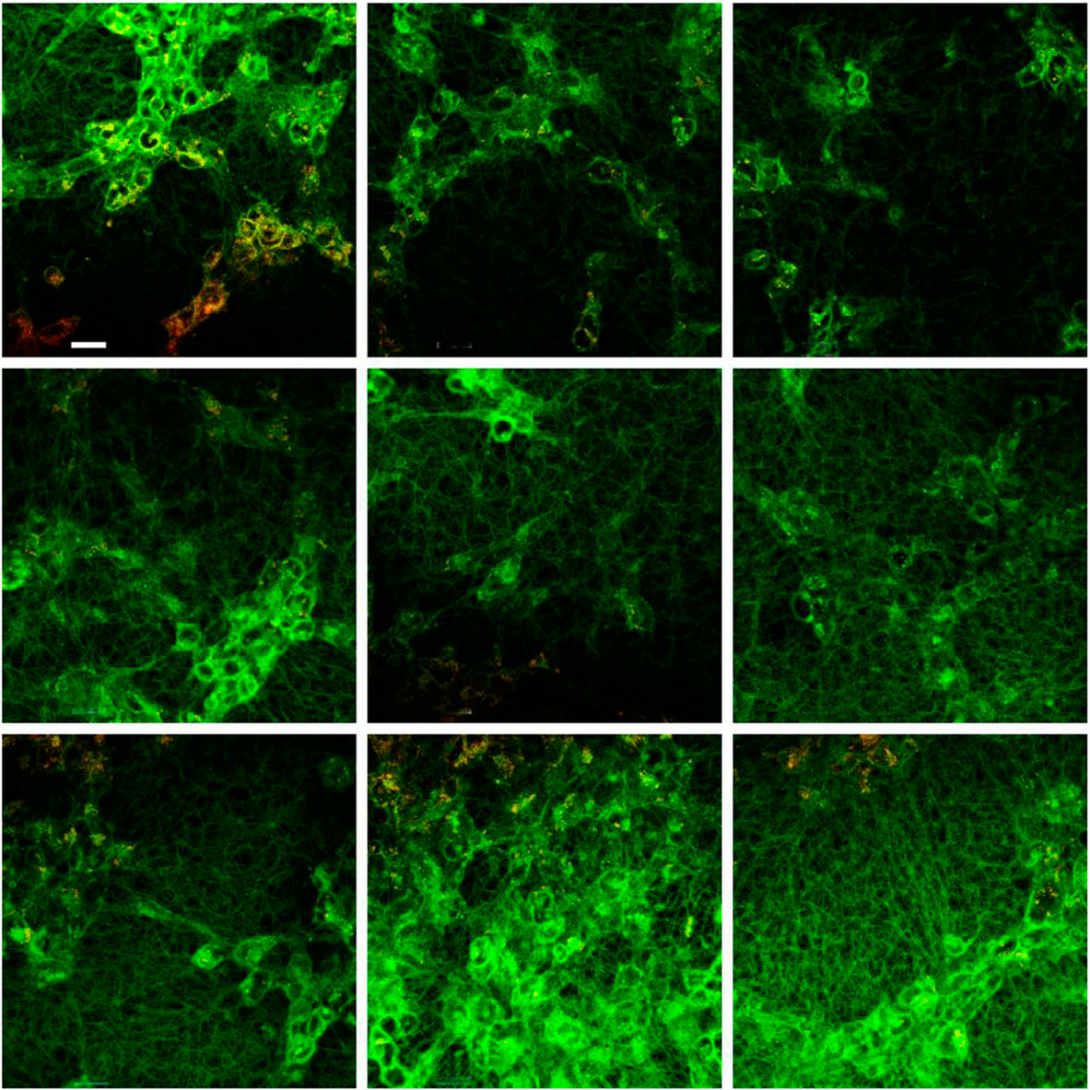
Fibrin 3% γ’ formed on the top of HMEC-1. Nine representative fields were selected at 10 μm from the cell surface. Fibrin fibers were labeled with Alexa 488-conjugated fibrinogen (green), and HMEC-1 cells with di-8-ANNEPS (red). Fibrin colocalizing with cell membrane appears yellow. The tool bar represents 30 μm

**Fig. 5:**
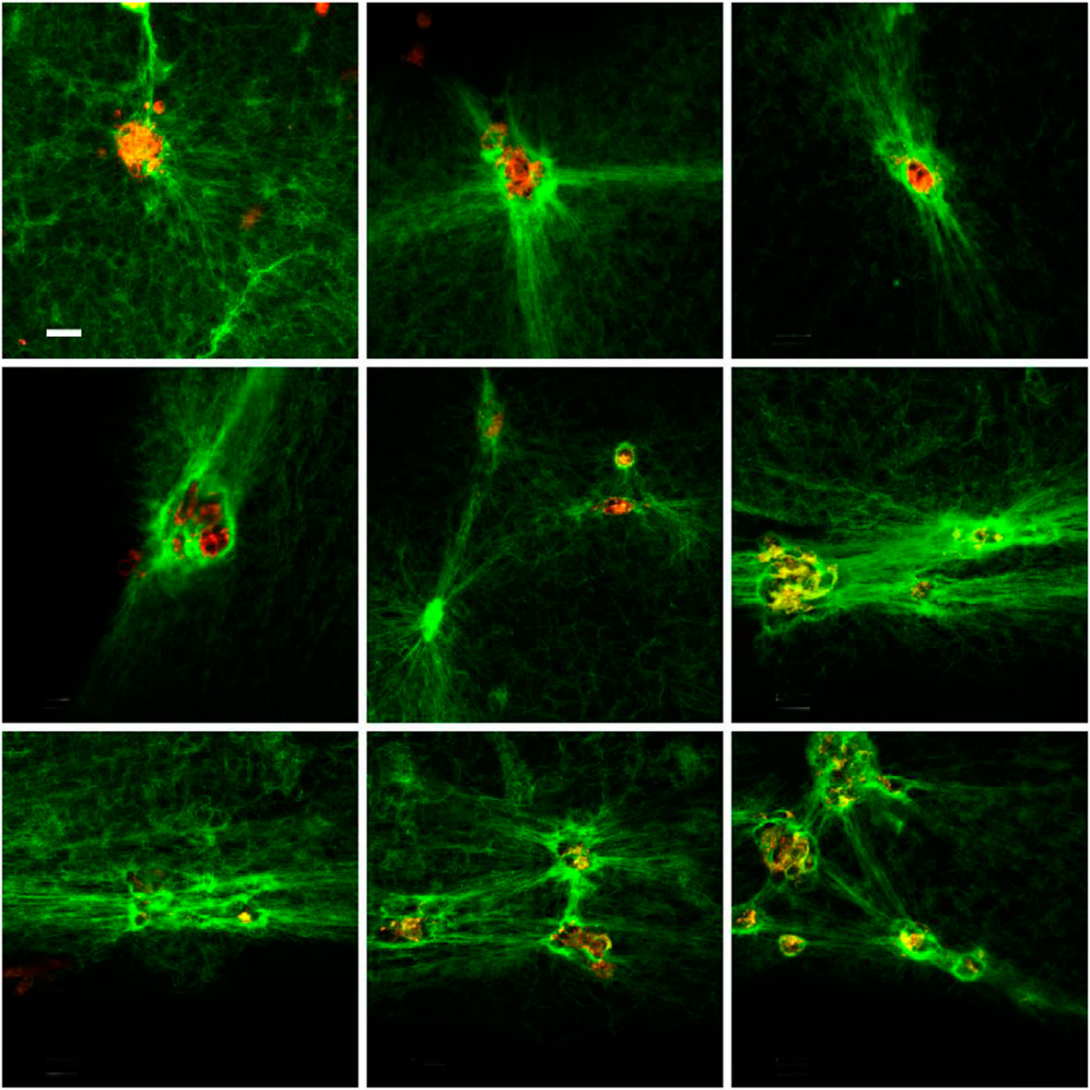
Fibrin 30% γ’ formed on the top of HMEC-1. Nine representative fields were selected at 10 μm from the cell surface. Fibrin fibers were labeled with Alexa 488-conjugated fibrinogen (green), and HMEC-1 cells with di-8-ANNEPS (red). Fibrin colocalizing with cell membrane appears yellow. The tool bar represents 30 μm

The interaction of the 3% γ/γ’ fibrin with HMEC-1 is shown in Figs. 4, and 30% γ/γ’ fibrin in Fig. 5. The most representative fields from different experiments were selected. As it can be clearly seen, fibers from 3% γ/γ’ fibrin were much less stressed in between cells compared to 30% γ/γ’ fibrin. At this γ/γ’ concentration, cells detached from the dish, and formed a round clump of cells surrounded by stressed fibers. The fibers of 30% γ/γ’ fibrin were appreciably thinner compared to 3% γ/γ’ fibrin.

## Discussion

In healthy individuals fibrinogen γ’ comprises 8–15% of total fibrinogen [27]. This minor fibrinogen variant has been found increased in arterial thrombosis and diminished in venous thrombosis [8], which has been the object of extensive research. However, this is the first time that the effects of fibrin (ogen) γ/γ’ on endothelial cells are reported.

The main finding of the present work is that fibrin (ogen) γ/γ’ levels alter the secretion of the fibrinolytic components uPA and PAI 1 thus mimicking the effect of thrombin. In order to imitate physiological conditions or to reach pathological levels of fibrinogen γ/γ’ described in the literature, this variant was mixed with fibrinogen γ/γ, differing from previous studies where either purified [20–22, 28] or recombinant [29, 30] fibrinogen γ/γ or fibrinogen γ/γ’ were used.

The effect of fibrinogen γ/γ’ on fibrin formation was studied at three different γ/γ’ concentrations: 3, 10 and 30%. At 3 and 10% the kinetics of fibrin formation were similar. In contrast, at 30% γ/γ’ the slope and final turbidity decreased approximately 2-fold, in the absence of cells. However, when clots were formed on the top of HMEC-1 cells, the differences were more subtle (1.7 and 1.3, respectively). Cooper et al. [20] using 100% fibrinogen γ/γ and fibrinogen γ/γ’, purified from plasma, obtained similar results (2-fold differences in final turbidity; the slope of the curves were not reported), and Gersh et al. [30] working with recombinants fibrinogen, reported a decrease of the slope and final turbidity of 2 and 1.5-fold, respectively. It is noteworthy that apparently, a content of 30% of fibrinogen γ/γ’ induced a similar effect in the kinetics of fibrin formation to that of 100% fibrinogen γ/γ’.

Thrombin has a spectrum of effects on different cell types including platelets, endothelial cells, and fibroblasts [31]. In endothelial cells, thrombin enhances the synthesis of tissue type plasminogen activator (tPA) and its inhibitor, plasminogen activator inhibitor type 1 (PAI 1). We investigated the effects of the γ/γ’ fibrin content on the secretion of fibrinolytic components by HMEC-1 cells. It has been previously demonstrated that HMEC-1 cells synthesize PAI 1 and uPA but not tPA [32–34].

Since the kinetics of fibrin formation with 3 and 10% of γ/γ’ were similar, it was decided to compare a low and high fibrin content of γ/γ’ of 3 and 30%, respectively. When cells were incubated with 1 nM thrombin alone (the same quantity used to form clots on the top of the cells) or 30% of γ/γ’, the secretion of uPA was increased 2-times as compared to the basal condition and 3% of γ/γ’, indicating that 30% of γ/γ’ clot’s content had the same effect as thrombin alone to activate this microvasculature cell line. The uPA concentrations in the cell lysate were greater than those found in the supernatant in all stimulatory conditions. Probably at the time that the cells were lysed (after 12 h of incubation) the majority of uPA was not secreted to the extracellular medium, or recaptured by its receptor. In addition, the incubation of the cells with 30% of γ/γ’ produced the highest amount of uPA inside the cells. Like uPA, the PAI 1 concentration was higher in the presence of thrombin and 30% of γ/γ’, both in the supernatant and cell lysate. But in contrast to uPA, the intracellular PAI 1 concentration was diminished in the cell lysate compared with the supernatant (in between 4.5 and 3-time less, respectively). Since the γ’ chain binds thrombin through exosite II [35], increasing the γ/γ’ content of the clot the quantity of thrombin bound to fibrin would consequently be augmented, concentrating thrombin on the cell surface. Therefore, the cellular effects observed with fibrinogen γ/γ’ can be attributed to the thrombin bound to the γ’ chain, and not to the fibrinogen γ/γ’ per se*.*

Several groups have reported the effects of fibrinogen γ/γ’ on clot structure, including diminished fiber diameter, increased fiber tapered ends, inhomogeneities in the network, i.e. areas with greater pore size, and recently it was published that changes introduced by fibrinogen γ/γ’ were due to its effects on protofibrils formation due to the repulsive forces between the highly negative charges content at the γ’-C-termini that impairs the D-D contacts when two γ’chains are aligned, shortening the length of the protofibrils, such that the network gelled earlier [36], and probably reflected in a prolonged lag time.

The present work has several limitations. First, the cell line used do not secrete tPA, in the future other cell line, as HUVEC or EA. Hy926, should be used for a more physiological or pathological simulation of in vivo situation. Furthermore, the consequences of the increased secretion of fibrinolytic components at high fibrin γ/γ’ should be tested.

Further investigations on cellular effects of fibrinogen γ/γ’ could shed light on thrombotic mechanisms related to its increase in arterial thrombosis or to its decrease in venous thrombosis.

## Conclusions

A concentration of 30% fibrin γ/γ‘ alter drastically fibrin structure on the cell surface, and affects the secretion of uPA and PAI 1 through its capacity to bind thrombin.

Probably, the deleterious effects of increasing fibrinogen γ/γ’ content in arterial thrombosis are related to the corresponding increase in the quantity of thrombin bound to the clot.

## Methods

### Fibrinogen purification

Plasma was supplemented with 200 U/mL aprotinin, 1 mM phenylmethylsulfonyl fluoride (PMFS), 5 mM etilendiaminotetracetic acid disodium salt (EDTA Na_2_), and applied to a Lysine-Sepharose® (Health Care, USA) column in order to remove plasminogen [37]. Then fibrinogen was precipitated with β-alanine, essentially as described [38]. Briefly, vitamin K- dependent proteins were adsorbed first with 2.4 mg/mL MgSO_4_ and then twice with 90 g/L BaSO_4_. The supernatant was precipitated thrice with 6 M β-alanine (2.7 M, final), with gentle stirring during 30 min at room temperature (RT), then centrifuged at 2000 g for 30 min at 4 °C. Each time the pellet was dissolved with Tris-saline buffer (TSB) pH 7.4 (50 mM Tris, 0.15 M NaCl) supplemented with 0.1 M epsilon aminocaproic acid (EACA). The pellet of the last precipitation was dissolved in TSB pH 7.4 and dialyzed overnight against the same buffer. The clottability of purified protein was > 90%. The purified fibrinogen was depleted of plasminogen, contaminated mainly with FXIII, and the integrity of the fibrinogen chains was confirmed by SDS/PAGE.

### Fibrin polymerization on the surface of HMEC-1 cell at different fibrinogen γ’ concentrations

#### Cell culture

MCDB131 medium (GIBCO, USA) supplemented with 10% foetal bovine serum (FBS) (GIBCO, USA), 100 U/mL penicillin (GIBCO, USA), 100 μg/mL streptomycin (GIBCO, USA), 200 mM L-glutamine (GIBCO, USA), and 10 ng/mL epidermal growth factor (Invitrogen, USA) was used to suspend and cultivate HMEC-1 cells.

The cells (100,000 cells/well) were seeded on 96-well microtiter plate (Thermo Scientific Nunc, USA), and cultivated overnight essentially as described elsewhere [39].

#### Clot formation on the top of HMEC-1 monolayer

In an eppendorf tube were added 143 μL purified fibrinogen 1 mg/mL (3% fibrinogen γ/γ’, quantified by ELISA [40]), 1 mg/mL 10 and 30% fibrinogen γ/γ’ (prepared by mixing commercial γ/γ and γ/γ’ fibrinogen, Enzyme Research Laboratories, USA), 40 μL MCDB 131 medium without supplements, and 17.5 μL bovine thrombin (Sigma, USA) – CaCl_2_ (1 nM and 2 mM, respectively, final concentrations), and the mixture immediately transferred on the top of the cells or directly to the bottom of the well (control clots without cells). The optical density (OD) was read every 2 min at 350 nm in an Infinite 200 M (Tecan, Vienna, Austria). Experiments were performed each time in triplicate in three independent experiments. The kinetic of fibrin formation was characterized by measuring the lag time (min), slope (mOD/min) and final turbidity (mOD).

### HMEC-1 uPA and PAI 1 secretion under different stimulation conditions

HMEC-1 cells were seeded (100,000 cells/well) on 96 wells plates. When the culture reached approximately 80% confluence, 100 μL of fibrinogen solution containing 3 and 30% of fibrinogen γ’, clotted with thrombin-CaCl_2_ (1 nM - 2 mM, respectively, final) was gently added on the cell surface. The plates were kept for 30 min in the incubator (5% CO_2_ in a moist environment), then 200 μL/well of supplemented medium was added and incubated again for 12 h. The control conditions were the cells cultivated during 12 h in the presence of medium with or without 10% FBS, or with thrombin – CaCl_2_ (1 nM - 2 mM, respectively).

The plates were centrifuged at 1000 g × 10 min and the supernatants (SN) were collected, aliquoted and stored at − 80 °C. The cells were lysed essentially as described [41]. The uPA and PAI 1 concentrations in supernatants and cell lysates were measured using uPA and PAI 1 ELISA kits (Sekisui-Diagnostic, Germany), according to the manufacturer’s recommendations. The ODs obtained were normalized dividing by the OD of the control condition of cells cultivated during 12 h without FBS. Each condition was tested in triplicate in three independent experiments.

### Determination of uPA receptor (uPAR) in HMEC-1

A citoELISA was performed in order to determine if HMEC-1 cells express uPAR. The assay is based on the assumption that if uPAR was present it will bind uPA, and uPA will cleave plasminogen to plasmin, and plasmin will cleave the Lys-pNA bond of S2251 substrate. Briefly, HMEC-1 cells were seeded (100,000 cells/well) on 96 well plates. When the culture reached approximately 80% confluence, the monolayer was washed three times with phosphate buffered saline (PBS) + 4 mg/mL albumin. Then, the monolayer of cells were incubated with 10 and 20 nM uPA (American Diagnostica inc. USA) for 1 h at 37 °C. After incubation, the cells were washed three times with PBS-albumin, 50 μL 0.5 μM plasminogen (American Diagnostica inc. USA) and 50 μL of 0.6 mM S-2251 (Chromogenix, Italy) were added. The plate was immediately transferred to a TECAN Infinite 200 M, and OD was read at 405 nm each 30 s during 1 h. The assay was performed in three independent experiments by duplicate.

### Fibrin interaction with HMEC-1

The procedure used was essentially as described previously [39]. Briefly, HMEC-1 cell membrane was stained with 4 μM di-8-ANEPPS (Molecular Probes, USA), and washed three times with 50 mM Tris, 0.15 M NaCl, pH 7.4 (supplemented with 20% MCDB-131 and 200 mM glutamine). Clots were formed on the cell surface by mixing 1 mg/mL fibrinogen (3 and 30% fibrinogen γ/γ’), supplemented with Alexa Fluor® 488 (Molecular Probes, USA)-conjugated fibrinogen, and thrombin-CaCl_2_ (1 nM and 1 mM, respectively, final). Polymerization was allowed to proceed for 2 h in the culture incubator, and then the clot structure on the surface of HMEC-1 was visualized using a Nikon Eclipse TE 2000-U laser microscope (with a 488 nm Argon or 543 nm HeNe laser). Image acquisition and processing were done as described elsewhere [39]. The quantification of the images was not included, since the measurement of fibrin density and width did not reflect the differences observed by LSCM.

### Statistical analysis

Results are expressed as mean ± standard error (SE). A two-way ANOVA was performed, with factor 1 as the experiment: clots without cells vs. clots with cells and factor 2, the different γ/γ’ fibrinogen concentrations. The effects of the different conditions used on uPA and PAI 1 secretion: controls and different γ/γ’ fibrinogen concentrations were analyzed by a one-way ANOVA, after a Shapiro-Wilk test for normality, Levene test for variance homogeneity, and a posteriori Tukey test. Furthermore, two way ANOVA analysis was performed in order to compare the differences between uPA and PAI 1 secretion at the different conditions used, and in the lysed cells. The SPSS 17.0 for windows (SPSS, Chicago, IL), and Origin Pro 8.1 software were used. A *p* < 0.05 was considered statistically significant.

## Data Availability

All data generated or analyzed during this study are included in this published article.

## Abbreviations

EACA: Epsilon aminocaproic acid
EDTA Na_2_: Etilendiaminotetracetic acid disodium salt
ELISA: Enzyme linked immunosorbent assay
FBS: Foetal bovine serum
HMEC-1: Human dermal microvascular endothelial cells
LSCM: Laser scanning confocal microscopy
OD: Optical density
PAI 1: Plasminogen activator inhibitor type 1
PBS: Phosphate buffered saline
PMFS: Phenylmethylsulfonyl fluoride
RT: Room temperature
SN: Supernatant
tPA: Tissue type plasminogen activator
TSB: Tris-saline buffer
uPA: Urokinase-type plasminogen activator
